# Spike Proteins of SARS-CoV-2 Induce Pathological Changes in Molecular Delivery and Metabolic Function in the Brain Endothelial Cells

**DOI:** 10.3390/v13102021

**Published:** 2021-10-08

**Authors:** Eun Seon Kim, Min-Tae Jeon, Kyu-Sung Kim, Suji Lee, Suji Kim, Do-Geun Kim

**Affiliations:** 1 Neuroimmunology Laboratory, Dementia Research Group, Korea Brain Research Institute, Sinseo, Daegu 41062, Korea; eunseon_k@kbri.re.kr (E.S.K.); jmt1986@kbri.re.kr (M.-T.J.); kks9286@kbri.re.kr (K.-S.K.); sujitmp2010@kbri.re.kr (S.L.); susie514@kbri.re.kr (S.K.); 2 Department of Brain & Cognitive Sciences, DGIST, Daegu 42988, Korea; 3 Department of Basic and Clinical Neuroscience, Institute of Psychiatry, Psychology, and Neuroscience, King’s College London, 16 De Crespigny Park, London SE5 8AF, UK

**Keywords:** COVID-19, SARS-CoV-2, BBB, endothelial cells, metabolism

## Abstract

Severe acute respiratory syndrome coronavirus 2 (SARS-CoV-2), which causes the coronavirus disease (COVID-19), is currently infecting millions of people worldwide and is causing drastic changes in people’s lives. Recent studies have shown that neurological symptoms are a major issue for people infected with SARS-CoV-2. However, the mechanism through which the pathological effects emerge is still unclear. Brain endothelial cells (ECs), one of the components of the blood–brain barrier, are a major hurdle for the entry of pathogenic or infectious agents into the brain. They strongly express angiotensin converting enzyme 2 (ACE2) for its normal physiological function, which is also well-known to be an opportunistic receptor for SARS-CoV-2 spike protein, facilitating their entry into host cells. First, we identified rapid internalization of the receptor-binding domain (RBD) S1 domain (S1) and active trimer (Trimer) of SARS-CoV-2 spike protein through ACE2 in brain ECs. Moreover, internalized S1 increased Rab5, an early endosomal marker while Trimer decreased Rab5 in the brain ECs. Similarly, the permeability of transferrin and dextran was increased in S1 treatment but decreased in Trimer, respectively. Furthermore, S1 and Trimer both induced mitochondrial damage including functional deficits in mitochondrial respiration. Overall, this study shows that SARS-CoV-2 itself has toxic effects on the brain ECs including defective molecular delivery and metabolic function, suggesting a potential pathological mechanism to induce neurological signs in the brain.

## 1. Introduction

Coronavirus disease 2019 (COVID-19) is infecting millions of people worldwide and has caused drastic changes in the lives of people [1]. The number of confirmed cases has exceeded two hundred millions worldwide, while more than four million deaths have occurred due to COVID-19 [2]. COVID-19 affects the respiratory system and its major symptoms include coughing, which can develop into severe pneumonia [3,4]. Severe acute respiratory syndrome coronavirus 2 (SARS-CoV-2), the infectious agent that causes COVID-19, is an enveloped RNA virus that belongs to the beta coronavirus [5]. SARS-CoV-2 is composed of four different structural proteins, including spike, envelope, membrane, and nucleocapsid protein [6]. Among these structural proteins, the spike protein has received the most attention, as it can induce potent immune responses and is a ligand for angiotensin converting enzyme 2 (ACE2), a receptor for SARS-CoV-2 [7,8]. The high infectivity of SARS-CoV-2 stems from the ability to bind to ACE2 much stronger than that of severe acute respiratory syndrome coronavirus (SARS-CoV) [9]. The spike protein bound to ACE2 is further cleaved into S1/S2 by cellular proteases, including cell surface, lysosomal, and extracellular proteases [7]. 

The blood–brain barrier (BBB) is a physicochemical barrier that constrains the entry of molecules and toxic substances into the brain [10,11]. It is composed of (ECs), pericytes, and astrocytic endfeet that limit the entry of molecules with molecular weights higher than 500 Da into the brain [12,13]. However, many infectious agents manipulate the machinery of the brain ECs to gain access to the brain [14,15]. Recent studies have reported that neurological signs are detected in patients with COVID-19 [16,17,18,19]. Brain ECs express ACE2, a receptor for the spike protein [20,21]. Endothelialitis from the peripheral system is observed in patients with COVID-19 [22]. This may indicate that the brain ECs may be the entry point of SARS-CoV-2 into the brain, and that it may damage the brain ECs during the invasion process [22,23]. Pathological insult on the brain ECs can induce changes in their normal physiology, which can potentially influence the molecular delivery to the brain reported in several types of diseases including neurodegenerative diseases [24,25]. Several studies reported that the regulation of transcytotic migration of molecules in the brain ECs is the major pathway in regulating molecular delivery, and that the disruption of their sophisticated control can induce dysfunction of the brain [26,27]. Moreover, changes in the metabolic status of ECs are key to the development of different diseases [28]. This is also applicable to the brain ECs where the changes in mitochondrial function can disrupt the integrity of the BBB, which may further trigger a more severe form of stroke demonstrated using a mouse model [29]. 

In this study, we focused on the pathological effect of spike protein on molecular delivery by affecting the endosomal trafficking pathway and metabolic signature of the brain ECs. In particular, we observed that the treatment of the spike protein to the brain ECs, which highly express ACE2, induces their internalization and changes in early endosomes in the bran ECs. Notably, these changes were dependent on the form of the spike protein, where the cleaved form of the spike protein, S1 domain (S1), enhanced the early endosomes. Meanwhile, the full-length form of the spike protein, active trimer (Trimer), exhibited the opposite effect. Similarly, altered transcytotic pathway was observed in the in vitro BBB system, suggesting that even S1 without viral particles can induce changes in the molecular delivery system in the brain ECs. We further conducted a live cell metabolic assay to examine the pathological effect of the spike protein on the mitochondrial function in the brain ECs. The results showed that the metabolic profile in mitochondria of the brain ECs is critically affected by the treatment with each individual spike protein, suggesting an important pathological role of the spike protein in the brain ECs. Our results demonstrate that the spike protein can induce dysfunction of molecular delivery and energy metabolism in the brain ECs by affecting dual pathological mechanisms.

## 2. Materials and Methods

### 2.1. Reagents and Antibodies

Primary and secondary antibodies were diluted at 1:500 for immunofluorescence as follows: anti-Rab5 (#3547, Cell Signaling, Danvers, MA, USA), anti-Spike S1 antibody (#100715-2, BPS, San Diego, CA, USA), anti-angiotensin converting enzyme 2 (ACE2, SC390851, Santa Cruz Biotechnology, Dallas, TX, USA), anti-TOMM20 (11802-1-AP, Proteintech, Rosemont, IL, USA), anti-rabbit IgG AF488 (#A32731, Invitrogen, Waltham, MA, USA), anti-mouse IgG AF568 (#A11004, Invitrogen), and anti-human IgG AF488 (#A11013, Invitrogen). The receptor binding domain (RBD, S1) of spike protein (BT10569, R&D Systems, Minneapolis, MN, USA) and active trimer of spike protein (10586-CV, R&D Systems) were purchased from R&D Systems.

### 2.2. Cell Culture

Human brain microvascular endothelial cells (HBMVECs) (ACBRI 376, Cell Systems) were cultured in a complete classic medium (#4Z0-500, Cell systems) with 2% culture boost (#4Z0-500, Cell Systems) supplemented with 1% Penicillin-Streptomycin solution (#15140-122, Gibco, Waltham, MA, USA). HBMVEC passage 5–12 cells were grown for 3–4 days at 37 °C and 5% CO_2_. All culture plates were coated with the attachment factor (#4Z0-500, Cell Systems) immediately before seeding the cells. Mouse brain endothelial cell lines (bEnd3, ATCC CRL-2299) were cultured in DMEM supplemented with 1% Penicillin-Streoptomycin solution and 10% fetal bovine serum (FBS, *Gibco*). bEND3 cells passage 20–23 cells were grown for 3–4 days at 37 °C and 5% CO_2_.

### 2.3. Immunofluorescence Assay (IFA)

HBMVECs (1 × 10^4^) were cultured for 3–4 days. After fixation with 4% paraformaldehyde, the cells were blocked with 5% normal goat serum (NGS)/PBS for 1 h at room temperature. Primary antibodies were diluted 1:500 in 0.5% BSA/PBS and incubated overnight at 4 °C. Secondary antibodies were diluted 1:500 in 5% NGS/PBS and incubated for 1 h at room temperature. The DAPI mounting solution was utilized for nuclear staining. The cells were imaged using a confocal microscope (TCS SP8, Leica, Wetzlar, Germany) at a magnification of 630×. The images were acquired at the Brain Research Core Facilities in KBRI. 

### 2.4. In Vitro BBB Permeability Assay

HBMVECs (1 × 10^4^) were cultured on 3.0 μm of porous membrane (353097, Corning, NY, USA) for 3 days. The media on the upper and bottom layers were replaced with fresh medium a day before the experiment. In the upper chamber, 10 μg/mL of AF488 conjugated transferrin (T13342, Thermofisher, Waltham, MA, USA) and 250 μg/mL of AF594 conjugated 10 kDa dextran (D22913, Thermofisher) were added in a time-dependent manner. At the indicated time points, the media from the bottom chamber were collected, and the fluorescence intensity of each fluorophore was analyzed using fluorometry (iD5, SpectraMax) with excitation at 491 nm and emission at 516 nm for AF488 and excitation at 590 nm and emission at 618 nm for AF488 conjugated transferrin and AF594-conjugated 10 kDa dextran, respectively.

### 2.5. Live-Cell Metabolic Assay

Seahorse XF kits were used to analyze the energy metabolism of the HBMVECs. To measure glycolysis, the Seahorse XF Glycolysis Stress Test (#103020-100, Agilent Technologies, Santa Clara, CA, USA) was performed. HBMVECs (1 × 10^5^) were seeded into an XF24 cell culture microplate (#102340-100, Agilent Technologies) and grown in the culture medium (as explained above) for 3–4 days at 37 °C and 5% CO_2_. The detailed procedure of the glycolysis stress test is described in the online user guide (#103020-100, Agilent Technologies). The Seahorse XF Mito Stress Test (#103015-100, Agilent Technologies) was performed to measure mitochondrial respiration. The culture conditions were the same as those described above. The detailed procedure of the Mito stress test is available in the online user guide (#103015-100, Agilent Technologies).

### 2.6. TUNEL Apotosis Assay

The HBMVEC cells were grown on coverslips and treated with spike protein (S1 and Trimer, at a concentration of 15 nM) for 24 h in order to analyze the apoptotic pathological effect of the spike protein. The cells were briefly washed with PBS and were fixed with 4% paraformaldehyde (PFA) for 15 min. Then, the cells were washed with PBS thrice and treated with ice-cold 70% ethanol for 30 min. The cells were subsequently incubated with a DNA labeling solution (2.25 μL of TdT enzyme, 30 μL of reaction buffer, 24 μL of BrdUTP, and 93.75 μL of dH_2_O) at 37 °C for 60 min. Then, the cells were washed with the rinse buffer and incubated with 1:20 diluted AF488 conjugated anti-BrdU antibody for 30 min at room temperature. At the end of the reaction, cells were further incubated with 0.5 mL of propidium iodide/RNase A staining buffer for an additional 30 min at room temperature. Finally, the cells were washed with the rinse buffer thrice and were analyzed using a confocal microscope (TCS SP8, Leica) acquired from the Brain Research Core Facilities in KBRI. Magnifications of 630× were used to acquire images of the mounted cells. 

### 2.7. Statistical Analysis

All experiments were performed in triplicate, data were analyzed using an unpaired two-tailed Student’s *t*-test followed by “D’Agostino-Pearson omnibus (K2)” normality tests to achieve data distribution. All data are expressed as a mean ± standard deviation (SD) using the GraphPad (v8.3.1) software. 

## 3. Results

### 3.1. Spike Protein of SARS-CoV-2 Enters the Brain ECs and Shows Strong Nuclear Localization

**Figure 1:**
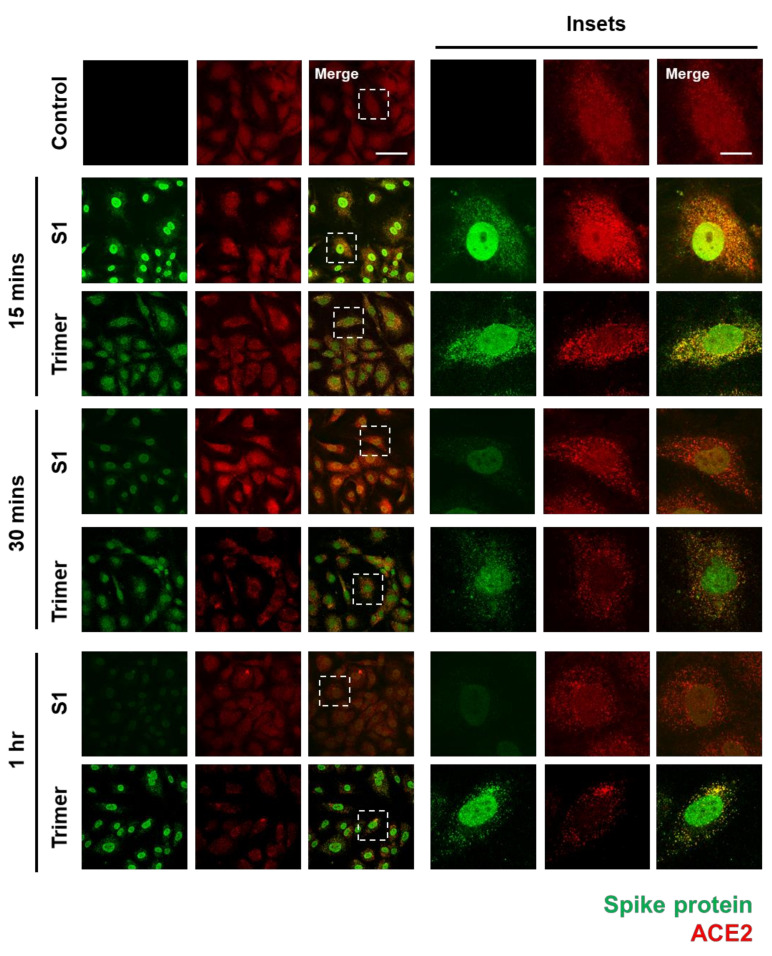
The spike proteins are internalized into the human brain ECs through ACE2. An amount of 15 nM of receptor binding domain (RBD) of spike protein (S1) and active trimer (Trimer) were treated on the primary human brain ECs for different time points up to 1 h, respectively. Cells were stained with spike protein (green) and ACE2 (red) antibody. Insets indicate magnified images of dashed lines. Scale bar, 75 μm (left panel); 20 μm (right panel).

To verify whether the spike protein of SARS-CoV-2 can enter the brain ECs, we used an immunofluorescent assay (IFA) to visualize its internalization into the brain ECs. First, we identified that ACE2 expression in the brain ECs was increased in the treatment with the spike protein compared to that in the control, especially at an early time point (15 min after treatment), and that nuclear localization of ACE2 was increased in both S1- and Trimer-treated brain ECs, respectively (Figure 1). We also observed that S1 is rapidly internalized into the brain ECs, and that S1 showed strong co-localization signals with ACE2 (Figure 1). This indicates that these two proteins, ACE2 and S1, interact with each other; this result is consistent with that of a previous report [9]. Notably, S1 protein was internalized and migrated into the nucleus, suggesting that it may regulate gene expression (Figure 1). Trimer internalization was also observed in the brain ECs, which also showed strong co-localization signals with ACE2. However, the localization of Trimer was not confined to the nucleus but rather distributed throughout the cytoplasm compared to that of S1. This suggests that the readily cleaved spike protein rapidly enters the nucleus, whereas the uncleaved form requires further processing to be delivered to the nucleus. This is an interesting phenomenon because the spike protein does not contain a nuclear localization signal, and it can potentially exert its role in gene regulation for effective cell invasion. Following experiments using sodium-azide that blocks the general molecular uptake also demonstrated a decreased entry of spike proteins in both S1 and Trimer treatment supporting the idea of their entry into the cell and nucleus (Supplementary Figure S1A). There are several reports showing that the ACE2 is not the only receptor for entry of SARS-CoV2 with utilization of spike protein [30,31]. To confirm the role of ACE2 in the internalization of the spike protein in the brain ECs, we have further performed experiments using mouse brain endothelial cell line (bEnd3) whose ACE2 is not binding to the spike protein of SARS-CoV2 [32]. We have observed that the both S1 and Trimer are not internalized in the mouse brain ECs indicating the important role of ACE2 in the internalization of spike proteins of SARS-CoV2 in the brain ECs (Supplementary Figure S1B).

### 3.2. Spike Protein of SARS-CoV-2 Activates Endosomal Trafficking Pathway in the Brain ECs

**Figure 2:**
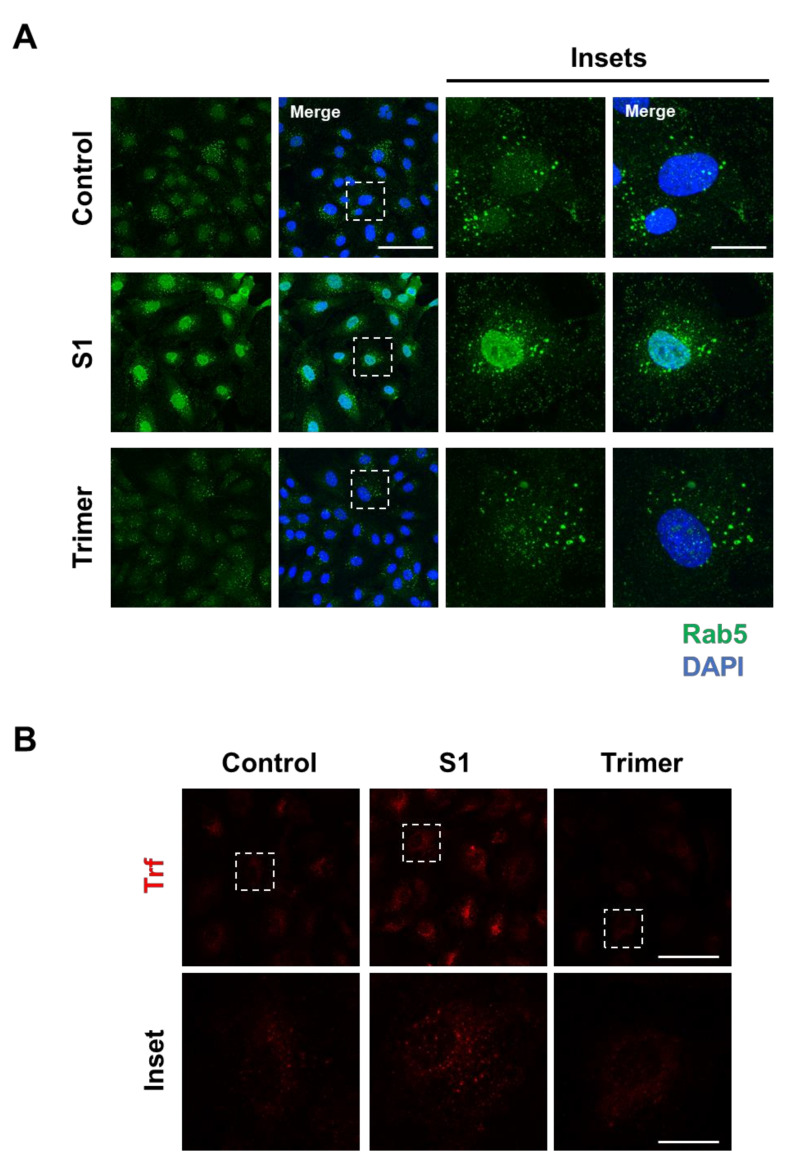
Endosomal trafficking is altered by the treatment of the spike proteins in the human brain ECs. (**A**) Human primary brain ECs were treated with 15 nM of S1 domain of spike protein (S1) or active trimer (Trimer) for 2 h, respectively. Cells were stained with Rab 5 (green). DAPI (blue) indicates nucleus. Insets indicate magnified images of squares of dashed lines. Scale bars 75 μm (left panel); 20 μm (right panel). (**B**) 10 μg/mL of human transferrin conjugated with Texasred (Trf, red) and 15 nM of S1 or Trimer were treated in the human primary brain ECs for 2 h, respectively. Scale bars, 75 μm (upper panel); 20 μm (bottom panel). Insets indicate magnified images of squares of dashed lines.

Brain ECs utilize the transcytotic pathway for effective delivery of molecules into the brain. This transcytotic pathway is tightly controlled through the endosomal-trafficking pathway to selectively allow the entry of molecules [33,34]. Therefore, disruption of transcytotic pathway can induce an increased influx of unwanted molecules or decrease essential molecular delivery into the brain, which is detrimental to the brain. To test whether the spike protein from the brain ECs can affect the endosomal trafficking pathway, we treated the spike protein to the brain ECs at different time points and observed the early endosome using a specific antibody against Rab5 (Figure 2A). We found that the treatment with S1 increased the level of Rab5 compared to the control (Figure 2A). In contrast, Trimer decreased early endosome formation, suggesting that molecular delivery in the brain ECs is suppressed (Figure 2A). In line with defective Rab5, to support the idea of altered molecular delivery mediated by the spike protein in the brain ECs, we performed transferrin (Trf) accumulation assay with the treatment of S1 or Trimer. These demonstrated that S1 treatment could induce increased accumulation of Trf, whereas Trimer decreased its accumulation (Figure 2B). 

**Figure 3:**
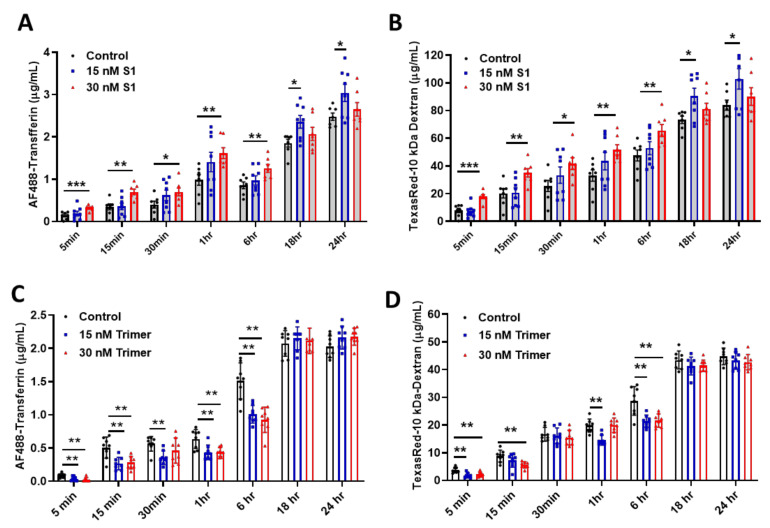
The permeability of molecular delivery is impaired by the treatment of the spike proteins in the human brain ECs. (**A**,**B**) Permeability tests were performed with the treatment with 15 nM (blue) or 30 nM (red) of S1 domain of spike protein (S1) each along with of human AF488-Transferrin (10 μg/mL) (**A**) and TexasRed-10 kDa-Dextran (250 μg/mL) (**B**) at different time points on the upper chamber. Data were quantified by unpaired student *t*-tests and means with SD (N ≥ 7). (**C**,**D**) 15 nM (blue) or 30 nM (red) of active trimer domain of spike protein (Trimer) was treated each along with AF488-Transferrin (10 μg/mL) (**C**) and TexasRed-10 kDa-Dextran (250 μg/mL) (**D**). Data were quantified by unpaired student *t*-tests, and error bars show means with SD (N ≥ 7). * *p* < 0.05; ** *p* < 0.01; *** *p* < 0.001.

As the brain ECs utilize the endosomal trafficking pathway to selectively regulate molecular delivery, we evaluated if the spike proteins can affect the delivery of molecules using an in vitro BBB model. We demonstrated that S1 treatment could induce enhanced permeability against human AF488-Transferrin (Figure 3A) and TexasRed-10 kDa Dextran (Figure 3B) molecules utilizing the transcytotic pathway in the brain ECs, compared to non-treated control. In contrast, the Trimer exhibited a suppressed level of human AF488-Transferrin (Figure 3C) and TexasRed-10kDa Dextran (Figure 3D) to the brain side. Thus, our data indicate that the two different species of the spike proteins feature different pathological roles of the brain ECs, especially during the molecular delivery.

### 3.3. Spike Protein of SARS-CoV-2 Induces Damage to the Mitochondria

**Figure 4:**
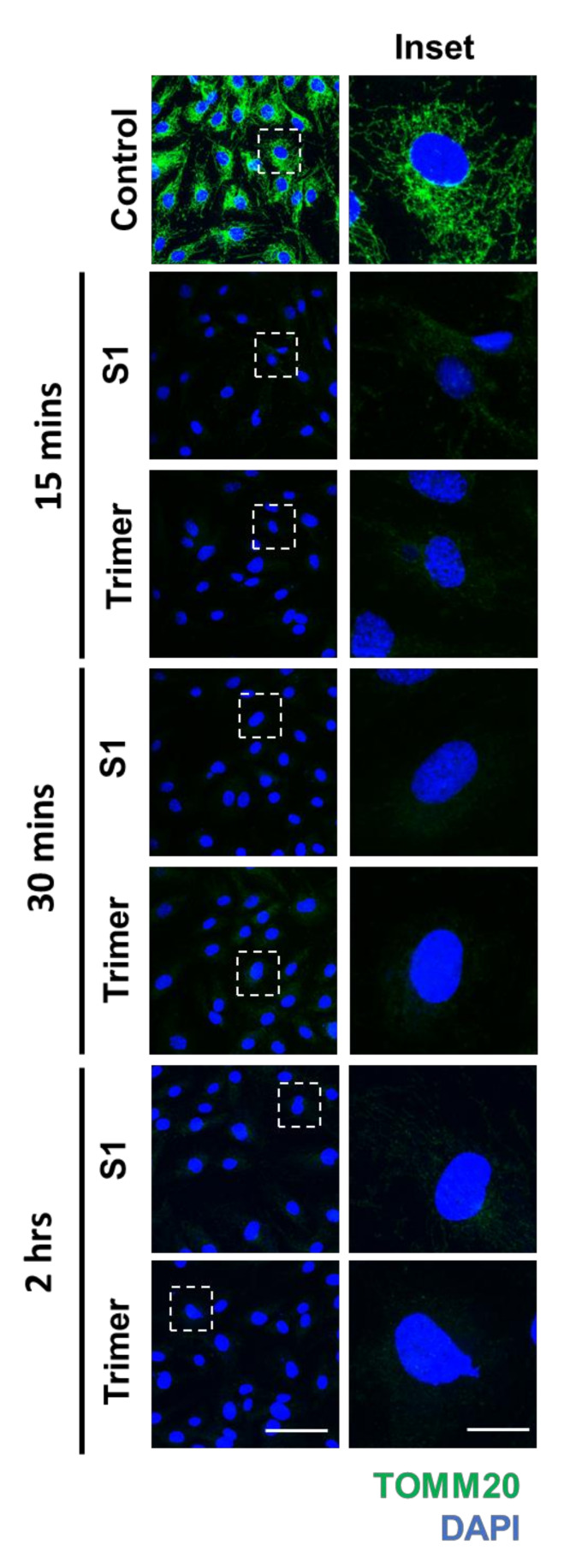
The spike proteins induce a decrease in the mitochondrial protein in the brain ECs. Primary human brain ECs were treated with 15 nM of S1 domain of spike protein (S1) and active trimer (Trimer) for 15, 30 min, and 2 h, respectively. Both are stained with antibody against translocase of the outer mitochondrial membrane complex subunit (TOMM) 20, a marker for the mitochondria (green). DAPI (blue) indicates nucleus. Insets indicate magnified images of squares of dashed lines. Scale bar, 75 μm (left panel); 20 μm (right panel).

Previous studies have shown that viruses can damage mitochondria to control the behavior of cells to facilitate further infection of cells [35,36,37]. A recent study has also reported that damage to the mitochondrial DNA (mtDNA) in the plasma is observed in severe cases of SARS-CoV-2 infection [38]. Disfunction in the mitochondria of endothelial cells can trigger diseases such as diabetes and cancer, emphasizing the important role of energy metabolism in the vasculature to maintain the normal physiology of organs [39,40]. These studies warrant the need to test the ability of the spike protein to induce mitochondrial damage and metabolic dysfunction. To this end, we performed IFA to determine the morphological changes induced by the spike protein. We identified that both S1 and Trimer of the spike proteins of SARS-CoV-2 could induce decreased signals of translocase of the outer mitochondrial membrane complex subunit (TOMM) 20, a marker for the mitochondria, beginning from 15 min to 2 h after S1 or Trimer treatment (Figure 4).

**Figure 5:**
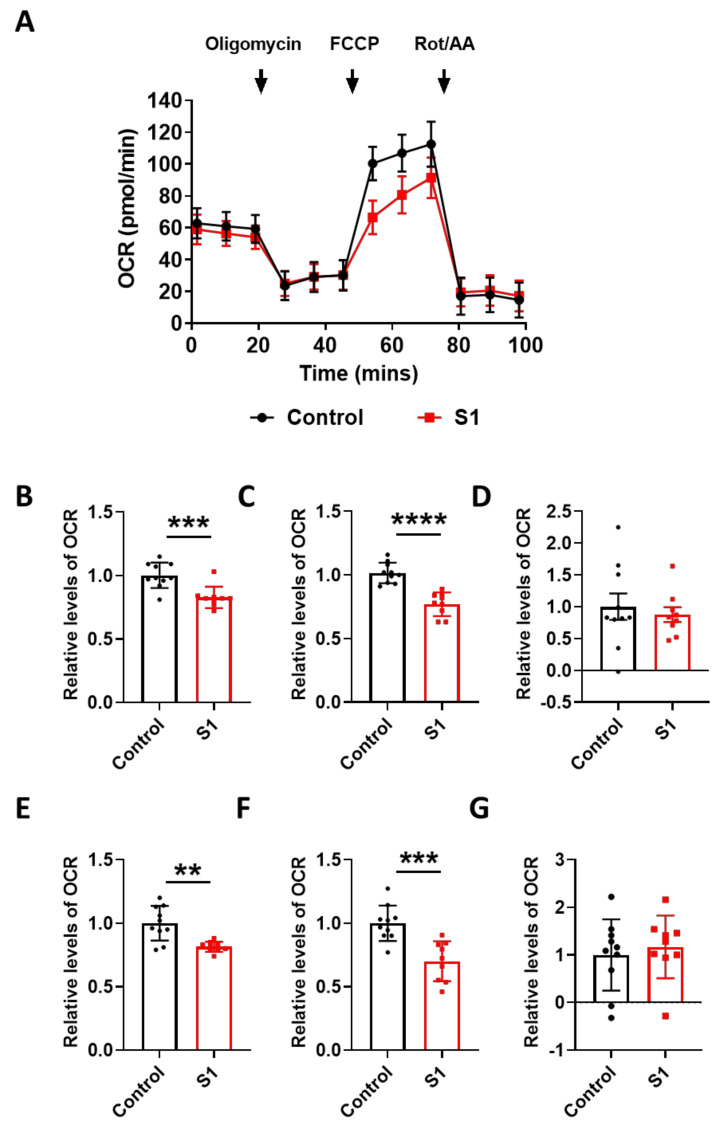
S1 domain spike protein reduces overall mitochondrial respiration of the human brain ECs. (**A**) Mito stress test was performed with the treatment of S1 (15 nM), followed by injection: 2.5 μM oligomycin; 2 μM FCCP; 0.5 μM Rotenone/Antimycin. (**B**–**G**) Parameters are calculated by Wave software program (*Agilent*) and normalized by nontreated control indicating basal respiration (**B**), maximal respiration (**C**), proton leak (**D**), ATP production (**E**), spare respiratory capacity (**F**), and non-mitochondrial respiration (**G**). Data were quantified by unpaired student *t*-tests, and error bars show means with SD (N ≥ 9). ** *p* < 0.01; *** *p* < 0.001, **** *p* < 0.0001.

**Figure 6:**
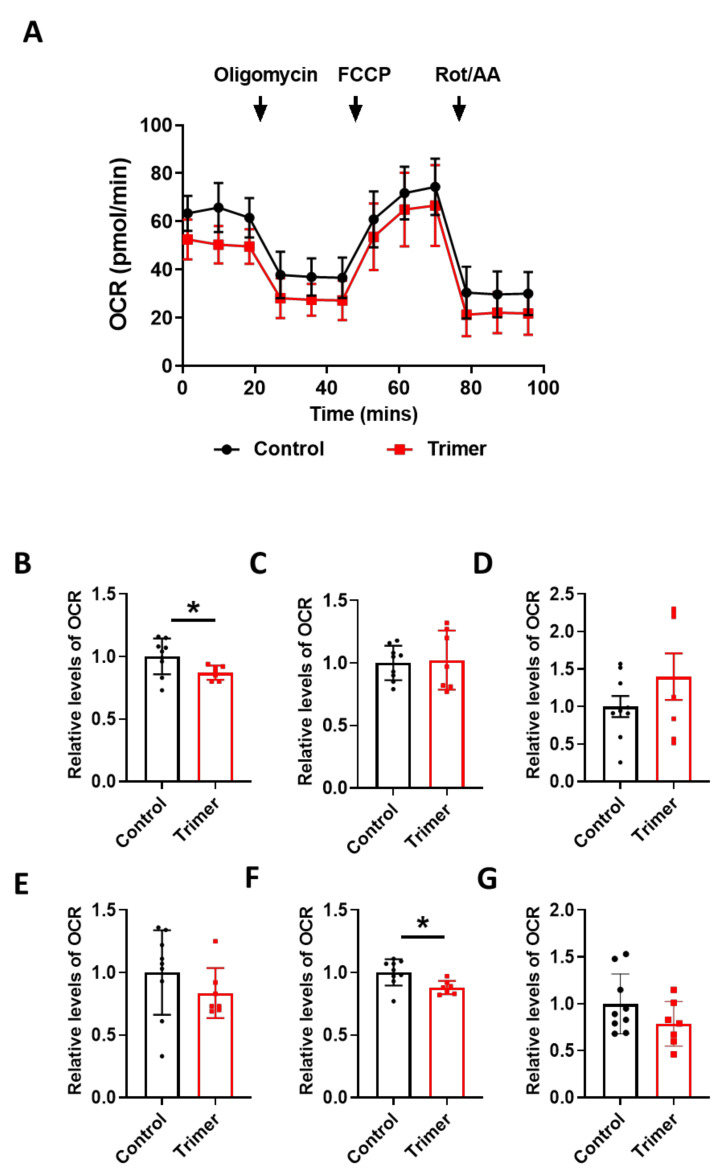
Active trimer spike protein alters basal respiration and ATP production of mitochondria in the human brain ECs. (**A**) Mito stress test was performed with the treatment of active trimer (Trimer) (15 nM), followed by injection: 2.5 μM oligomycin; 2 μM FCCP; 0.5 μM Rotenone/Antimycin. (**B**–**G**) Parameters are calculated by Wave software program (*Agilent*) and normalized by non-treated control indicating basal respiration (**B**), maximal respiration (**C**), spare respiratory capacity (**D**), proton leak (**E**), ATP production (**F**), and non-mitochondrial respiration (**G**). Data were quantified by unpaired student *t*-tests, and error bars show means with SD (N ≥ 7). * *p* < 0.01.

To find the link between the pathological damage to the mitochondria and functional deficits, we carried out a real-time metabolic assay to test the metabolic changes in the brain ECs. S1 treatment induced pathological damage to mitochondrial function (Figure 5A). Basal respiration of the brain ECs was significantly decreased in S1 treatment compared to the non-treated control (Figure 5B). Moreover, the maximum capacity, proton rate of the brain ECs was remarkably decreased in the treatment with Trimer compared to that in the non-treated control (Figure 6A), indicating that the spike protein can indeed induce pathological damage to the mitochondrial function in the brain ECs.

In particular, basal respiration and ATP production were largely decreased in Trimer treatment, whereas other indices were not severely affected. These suggest that the functional activity of the mitochondria was affected, but its activity upon energy demand was not significantly damaged. Overall, our results indicate that the spike protein from SARS-CoV-2 can induce mitochondrial damage to the brain ECs affecting their respiratory function, possibly leading to the neurological symptoms observed in patients with COVID-19. 

### 3.4. Spike Proteins of SARS-CoV-2 Do Not Induce Apoptotic Changes in the Primary Human Brain Endothelial Cell

**Figure 7:**
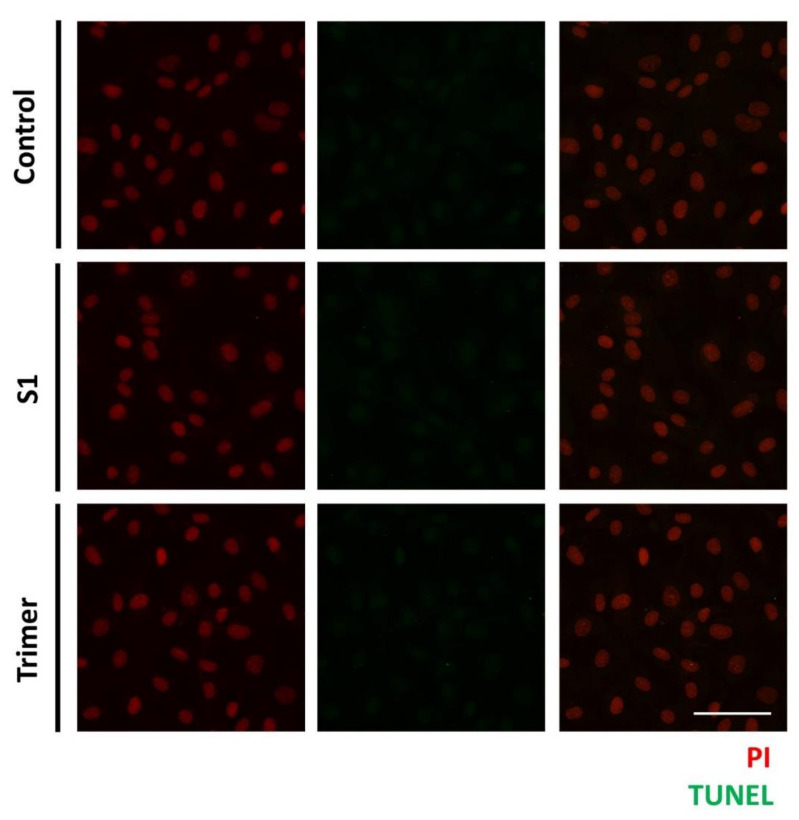
The spike proteins do not induce apoptosis in the human brain ECs. Human brain ECs were treated with 15 nM of S1 and active trimer for 24 h, respectively. DNA fragmentation, as the indicator for apoptotic changes, was assessed with Terminal deoxynucleotidyl transferase dUTP nick end labeling (TUNEL) assay. DNA fragmentation was labeled with AF488 conjugated anti-BrdU antibody (green), which was detected neither control nor the spike proteins (S1 and Trimer) treatment groups. Nucleus was counterstained with propidium iodide (PI, red). Scale bar, 75 μm.

As mentioned above, we observed the pathological defects in the human brain ECs, with the treatment of the spike proteins. Thus, to further verify whether the spike proteins from SARS-CoV2 can induce apoptotic changes in the brain ECs, we treated S1 and Trimer, respectively, to the brain ECs for 24 h and measured the DNA fragmentation of the nucleus using a TUNEL assay (Figure 7) [41]. We observed that both the S1 and Trimer did not induce apoptotic changes in the brain ECs, suggesting that physiological dysfunction, including molecular delivery and respiration defects induced by different types of spike proteins, do not induce cell death (Figure 7). 

## 4. Discussion

The COVID-19 pandemic is rapidly changing the environment that we live in owing to the high infectivity of SARS-CoV-2. Currently, more than two hundred million people are infected, and the total number of deaths exceeds four millions [2]. Several studies report that COVID-19 can induce brain damage, including microhemorrhages, headaches, and confusion [42]. Moreover, as the number of patients is increasing, these patients may seemingly experience neurological problems for a longer period of time [43]. Similar neurological signs were observed in patients with acquired immunodeficiency syndrome (AIDS), including dementia-like symptoms [44]. The brain is one of the most desirable organs to hide for most pathogens because it lacks the adaptive immune system due to the existence of the BBB [45,46]. Therefore, pathogenic microbes utilize various approaches to gain access to the brain, and the first hurdle to overcome is the BBB [46]. The BBB protects the brain from peripheral factors that can potentially induce inflammatory signs [3,10]. Moreover, several transporters regulate the entry of molecules required for the survival and normal function of neuronal cells [10,33]. When a pathogen manipulates the machinery of the BBB to maximize the gain of entry into the brain, it is unavoidable to affect the normal function of the BBB. Although many studies have focused on its role in the viral entry of SARS-CoV-2 and the targets of immunogen for vaccine development, its pathological role in the brain has not sparked considerable interest. Thus, it is important to unravel the pathological effects of the spike protein on the BBB for several reasons. First, ACE2 protein is expressed in the brain ECs, and it is well-known that the ACE2 protein is the receptor for the spike protein of SARS-CoV2 [21,43]. This suggests that the brain ECs may be used as the portal for its efficient entry into the brain in the point view of SARS-CoV2. If the spike protein itself can induce pathological effects, entry of the virus in the brain ECs itself may trigger damage to the brain ECs. Second, most vaccines for COVID-19 are designed based on the spike protein [47]. If the spike protein itself has a high pathological effect on the brain vasculature, it may be of note to have preventative measures to minimize vasculopathy to limit the consequent damage to the brain after vaccine administration. 

Thus, we aimed to identify the pathological consequences of the spike protein on the brain ECs. We identified the existence of ACE2 protein, the receptor for the viral entry, which was co-localized with the spike protein (Figure 1). Internalized spike proteins are found to localize to the nucleus and cytoplasm (Figure 1). Notably, we observed that the spike protein in the nucleus does not contain the nuclear localization signal (NLS) in its peptide sequence. This itself may have pathological function or affect the modulation of cellular machinery, which may require further study to unravel its pathological effect. Recent studies have reported that the brain ECs regulate molecular delivery through the endosomal–lysosomal trafficking system to selectively allow the entry of molecules into the brain [33,34]. The spike protein induced a rapid increase in Rab5, a marker for the early endosome that is utilized for molecular recirculation (Figure 2A). Notably, some portion of Rab5 was also localized in the nucleus (Figure 2A); however, its function in the nucleus is not well understood. This phenomenon was distinct in the S1 portion of the spike protein compared to that in the Trimer complex (Figure 2A). Moreover, the increased early endosome was correlated with an elevated accumulation of transferrin utilizing the transcytotic pathway by the treatment of the spike protein (Figure 2B). Furthermore, we performed a permeability assay on brain ECs given the potential pathological effect of the spike protein on the function of brain ECs. The S1 domain of the spike protein induced increased permeability, whereas the Trimer decreased permeability (Figure 3). The mechanism of the effect of two subdomains on the permeability requires further in-depth studies.

Mitochondria are the powerhouse of cells, and some viruses are known to regulate mitochondrial function to increase the likelihood of infection [39]. Recent studies have shown that elevated levels of circulating mitochondria increased the severity of SARS-CoV-2 infection, emphasizing the correlation between mitochondrial damage and COVID-19 severity. Moreover, the brain ECs contain higher levels of mitochondria than peripheral vascular cells, possibly owing to the higher energy demand [29,48]. This suggests that the invasion of SARS-CoV-2 into the brain ECs would damage their normal function, especially their engagement in the molecular delivery system, which is essential for maintaining brain homeostasis. We found that the mitochondria of the brain ECs were critically affected by spike protein treatment (Figure 4). Moreover, the metabolic analysis disclosed that the spike protein also affects the function of the mitochondria in the the brain ECs, suggesting that they can affect the metabolic status of the brain ECs as well. The metabolic signature of the brain ECs between S1 and Trimer treatment was somewhat different in some metabolic indicators (Figure 5 and Figure 6). More specifically, S1 impaired broad lines of parameters in mitochondria rather than Trimer did, thus, emphasizing that the direct entry of S1 into the brain ECs is more pathogenic in terms of mitochondrial function than Trimer, indicating the requirement of the further cleavage for an effective entry (Figure 6). Several studies reported that the metabolic status of ECs is highly significant in the maintenance of organ homeostasis. The spike protein also decreases the activity of mitochondria and potentially disrupts the brain homeostasis. 

The ability of the spike protein to induce changes in BBB permeability provides several insights into the invasion strategy of SARS-CoV-2 that would modulate the permeability to peripheral molecules. In turn, it would also enhance the accessibility of SARS-CoV-2 into the brain. This can hinder the normal physiology of the brain ECs and affect the molecular delivery to the brain or induce vascular damage that would partially explain the neurological symptoms observed in patients with COVID-19. Increasing evidence suggests that SARS-CoV-2 infection can cause neurological deficits, including delusions, headaches, dizziness, and acute cerebrovascular diseases [1,16,49]. Although the mechanisms by which SARS-CoV2 can induce various types of neurological symptoms remain elusive, our results prove that the spike protein itself can induce BBB dysfunction, partially explaining the neurological signs induced by SARS-CoV2. We believe that the BBB dysfunction induced by internalized spike protein may indirectly cause detrimental effects on neuronal cells, or that the spike protein crossing the BBB may enter neuronal cells, leading to the toxic effects. However, the experimental evidence on this topic is still scanty and, thus, requires further research. Our future studies will focus on examining the neurological pathology induced by the spike protein using an animal model and on revealing its correlation with the BBB dysfunction. Another important topic of study required is the effect of mutations in the spike protein and its impact on the pathological effect on the brain ECs. For example, D614G mutation shows higher levels of ACE2 binding affinity compared to the wild type of the spike protein of SARS-CoV2 [50]. It is speculated that the mutation of the spike protein is the evolutionary strategy to infect the host cells more efficiently, and that it will enhance the binding affinity of the ACE2 in the brain ECs that will potentially increase the infectivity of SARS-CoV2 and pathological effect on the brain ECs. Considering its importance in the pathogenicity of the brain ECs and its neurological effect, we are planning to dissect this topic in the near future.

Moreover, our data can also provide valuable information for the assessment of safety in COVID-19 vaccines because most of them utilize the spike protein as an epitope to induce immune responses. Our study provides the arguments that the spike protein can induce changes in the normal physiology of the brain ECs. Moreover, several reports have shown the adverse effects of vaccines, including anaphylactic shock, which could be related to vasculopathy [51,52]. Moreover, a report describing neurological signs, including Guillain–Barre syndrome (GBS), a rare type of autoimmune disease attacking myelin induced by vaccine administration, potentially supports our hypothesis [53]. Recently, the spike protein itself can induce a toxic effect on the lung endothelial cells that has focused on the metabolic changes and barrier integrity changes [54]. Additionally, the effect of the spike protein on brain ECs that mostly focused on the paracellular permeability and inflammatory responses induced by the spike protein administration [55]. Our study demonstrates that the spike protein can induce changes in the transcellular pathway, which is one of the most crucial pathways governing molecular delivery to the brain and mitochondrial dysfunction. These are expected to have a key role in the normal brain function that analyzed the pathogenicity of the spike protein in different angles focusing on the brain ECs. Although a direct correlation between the BBB dysfunction and the occurrence of neurological symptoms is lacking, it would be an important topic for future studies. Our study suggests that the spike protein-based vaccine could potentially be harmful to the brain ECs, and further analysis of the neurological function by vaccine administration might need to be performed to validate the safety of the vaccine.

It is still elusive how SARS-CoV2 is interacting with cellular components that are critical in understanding the pathophysiology of SARS-CoV2. Recently, several studies showed that the SARS-CoV2 virus can induce activation of autophagy, suggesting that the interaction of SARS-CoV2 with autophagic components can increase its capability of replication in host cells [30,56]. This is a good example demonstrating that host–pathogen interaction may be one of the driving factors inducing the pathology observed in the host rather than the innate toxic effect of viral components [56]. Similarly, it is important to dissect precise molecular mechanisms from the host side elicited by the spike proteins to better understand the pathophysiological phenomena from our study to find a therapeutic plan. However, experimental evidence supporting molecular mechanisms behind are still lacking, thus it will be an emerging topic of our future study.

## 5. Conclusions

This study has focused on the pathological roles of the spike proteins on the disruption of normal function in the brain ECs. We observed that the different types of the spike proteins (S1 and Trimer) can be internalized into the brain ECs and can mediate changes in the delivery of molecules. Additionally, these proteins could induce the functional defects in the mitochondria that may be linked to the function of the brain ECs. Accumulating evidence is suggesting that the SARS-CoV2 infection may induce subsequent neurological signs. Even though we do not have direct evidence that the observed damage to the brain ECs by the spike proteins can induce neurological signs in the brain, this study may provide important information to find the link between the spike protein-mediated brain vascular damage and the development of neurological signs that could be the topic of our future study.

## Data Availability

The data that support the findings of this study are freely available at Zenodo at http://doi.org/10.5281/zenodo.4815661 (accessed on 21 May 2021).

## Supplementary Materials

The following are available online at https://www.mdpi.com/article/10.3390/v13102021/s1, Figure S1: Internalization of the spike proteins is blocked with sodium azide treatment and in mouse brain ECs. (**A**) Primary human brain ECs were pretreated for 30 min with 10 mM of sodium azide, a blocker for general entry of molecules into cells, and 15 nM of receptor binding domain (RBD) of spike protein (S1) and active trimer (Trimer) were treated on the primary human brain ECs for 15 min. Cells were stained with anti-spike protein antibody (green). Insets indicate magnified images of squares of dashed lines. Scale bar, 75 μm (upper panel); 20 μm (bottom panel). (**B**) bEND3 cells, mouse brain ECs lines, whose ACE2 is known not to bound to spike proteins, were treated with 15 nM S1 and Trimer for 15 min. Cells were stained with anti-spike protein antibody (green). Insets indicate magnified images of squares of dashed lines. Scale bar, 75 μm (left panel); 20 μm (right panel).
