# Supplementary material for: Spike Proteins of SARS-CoV-2 Induce Pathological Changes in Molecular Delivery and Metabolic Function in the Brain Endothelial Cells
Source: Viruses. 2021 Oct 8;13(10):2021. doi: 10.3390/v13102021 (PMC8538996; doi:10.3390/v13102021)
Supplement: Supplementary file 1 [file viruses-13-02021-s001.zip › viruses-1365631-supplementary.pdf]

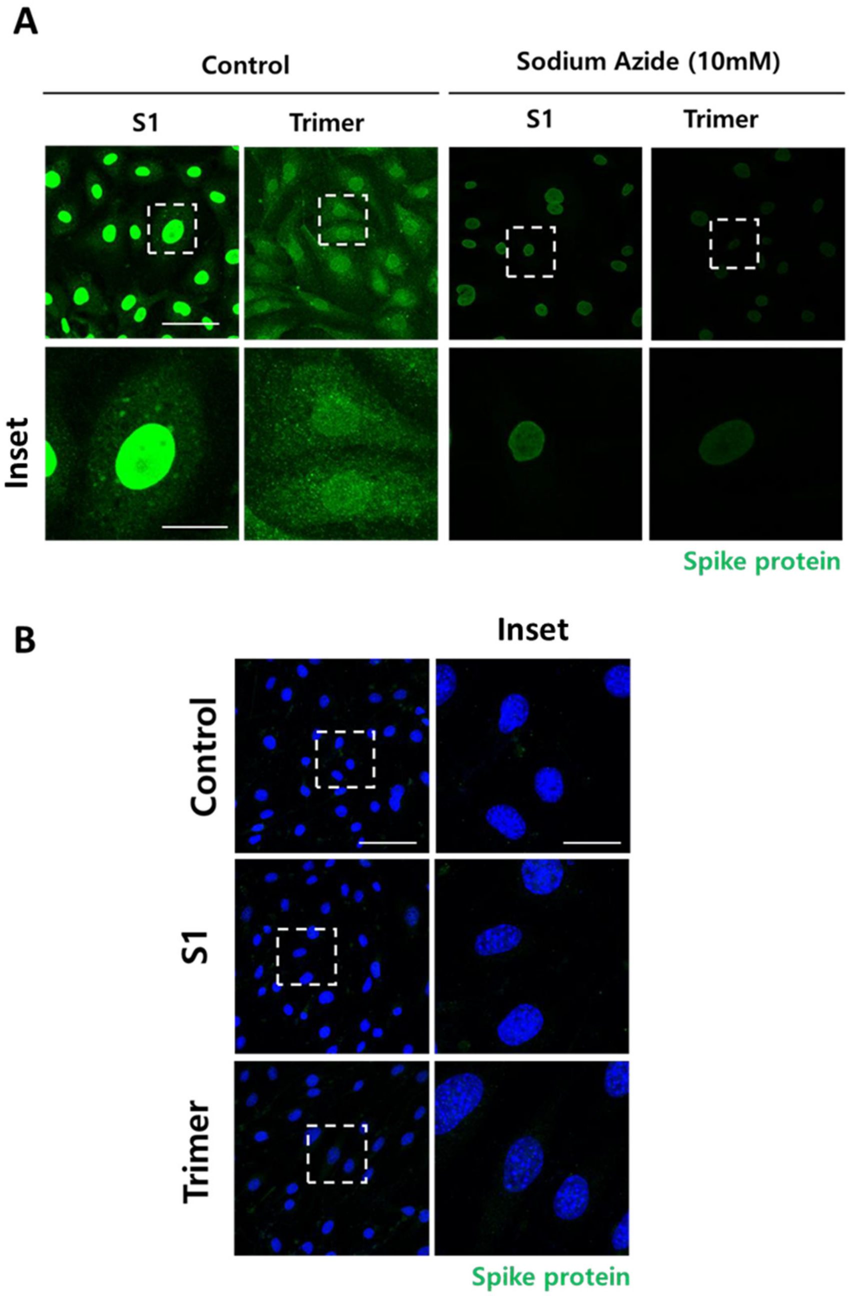

Figure S1: Internalization of the spike proteins is blocked with sodium azide treatment and in mouse brain ECs. **(A)** Primary human brain ECs were pretreated for 30 min with 10 mM of sodium azide, a blocker for general entry of molecules into cells, and 15 nM of receptor binding domain (RBD) of spike protein (S1) and active trimer (Trimer) were treated on the primary human brain ECs for 15 min. Cells were stained with anti-spike protein antibody (green). Insets indicate magnified images of squares of dashed lines. Scale bar, 75 μm (upper panel); 20 μm (bottom panel). **(B)** bEND3 cells, mouse brain ECs lines, whose ACE2 is known not to bound to spike proteins, were treated with 15 nM S1 and Trimer for 15 min. Cells were stained with anti-spike protein antibody (green). Insets indicate magnified images of squares of dashed lines. Scale bar, 75 μm (left panel); 20 μm (right panel).
